# The Effects of Trunk Intervention on Gross Motor Function, Balance, and Spasticity in Cerebral Palsy: Systematic Review and Meta-Analysis

**DOI:** 10.3390/medicina61081324

**Published:** 2025-07-23

**Authors:** Mi-Soo Lim, Byung-Chan Yoo, Hyoung-Won Lim

**Affiliations:** 1 Department of Physical Therapy, Graduate School, Dankook University, Cheonan 31116, Republic of Korea; altn6213@naver.com; 2 KCS Orthopedic Clinic, Seoul 08792, Republic of Korea; 3 Department of Physical Therapy, College of Health Sciences, Dankook University, Cheonan 31116, Republic of Korea

**Keywords:** cerebral palsy, trunk intervention, gross motor function, meta-analysis

## Abstract

*Background and Objectives:* Cerebral palsy (CP) is a non-progressive neurological disorder characterized by motor impairments such as spasticity and poor postural control. Among these, trunk control plays a critical role in maintaining balance and enabling functional mobility. Since spasticity is known to interfere with motor coordination and posture, evaluating its response to trunk-focused interventions may offer additional clinical insights. This systematic review and meta-analysis evaluated the effectiveness of trunk-focused interventions on trunk control, gross motor function, balance, and spasticity. *Materials and Methods:* A systematic search was conducted in PubMed, Embase, Web of Science, MEDLINE, and CINAHL for randomized controlled trials (RCTs) published in the last 10 years up to 11 April 2023. Studies targeting trunk-specific interventions in children with CP were included. Meta-analyses were performed using RevMan 5.3, calculating standardized mean differences (SMDs) with 95% confidence intervals (CIs). Study quality was assessed using the PEDro scale. *Results:* Fifteen RCTs involving 454 children were included. Trunk control improved significantly (SMD = 3.67; 95% CI: 3.10–4.25; I^2^ = 0%). Gross motor function showed a small but significant improvement (SMD = 0.49; 95% CI: 0.06–0.92; I^2^ = 44%). Balance exhibited a large, though not statistically significant, effect (SMD = 0.90; 95% CI: −0.00 to 1.79; I^2^ = 81%). Subgroup analysis indicated that interventions performed more than four times per week produced a significant effect on balance (SMD = 0.54; 95% CI: 0.08–1.01). Only one study assessed spasticity and found no group difference. *Conclusions:* Trunk-based interventions significantly improve trunk control and gross motor function in children with CP. While improvements in balance were inconsistent, higher-frequency interventions yielded more favorable results. Further research is warranted to clarify effects on spasticity and optimize intervention protocols for clinical application.

## 1. Introduction

Cerebral palsy (CP) is a non-progressive neurological disorder resulting from early brain damage, leading to lifelong motor and postural impairments that significantly restrict daily activity and social participation [1]. According to the World Health Organization (WHO), strength-based interventions delivered by qualified physical and occupational therapists are essential for improving motor skills and mobility in children with CP [2].

Among the various motor impairments associated with CP, deficits in trunk control are particularly critical, as the trunk provides postural stability and coordinates limb movements [3]. Effective trunk control enables anticipatory postural adjustments—essential for sitting, standing, and walking [4,5,6]—and its impairment is linked to reduced independence in daily activities and a lower quality of life [6].

Growing evidence suggests that strengthening trunk muscles enhances motor precision and functional performance in children with CP. Trunk control has been identified as a strong predictor of gross motor function, balance, and gait ability [3,5,7]. For instance, Balzer et al. [7] demonstrated that trunk stability influences walking capacity through its interaction with lower-limb spasticity and selective motor control. Notably, spasticity—a common motor impairment in CP—interacts with trunk control and lower-limb function, and although its direct impact on gait and gross motor function may be modest, it remains clinically relevant within the broader spectrum of motor impairments [7]. These findings highlight trunk function as a pivotal element in postural organization and balance regulation during childhood development [8].

Furthermore, recent studies have emphasized that trunk control is not only essential for postural and movement coordination [9,10], but also closely related to self-care independence and overall functional mobility [11]. Wallard et al. [12,13] and Pierret et al. [14,15] reported that children with CP experience significant difficulties in stabilizing the head and trunk during movement, which adversely affects gait and daily functioning. These findings underscore the clinical importance of evaluating and addressing trunk control in pediatric rehabilitation.

Clinically, the International Classification of Functioning, Disability and Health (ICF) framework categorizes trunk function within the domain of body structures and functions, highlighting its relevance to both physical capacity and rehabilitation outcomes [16]. Trunk-centered interventions—including motor control training, coordination exercises, and task-specific therapy—have shown promise in improving strength, postural control, and coordination in children with CP [16,17,18,19]. These approaches have also been associated with enhancements in functional outcomes such as balance, sitting ability, and gait. However, despite the growing body of research, variations in intervention types, outcome measures, and methodological quality make it difficult to draw consistent clinical conclusions.

While several recent systematic reviews have evaluated trunk-focused interventions in children with cerebral palsy (CP), none have synthesized randomized controlled trials (RCTs) encompassing the full spectrum of functional outcomes—namely, trunk control, gross motor function, balance, and spasticity. For instance, Mohamed et al. [20] demonstrated that a 12-week core stability program significantly improved standing ability, balance, and gait parameters in children with mild spastic diplegic CP compared to conventional physiotherapy. Similarly, a single-blinded RCT included in this review reported that task-oriented activities based on neurodevelopmental therapy principles led to significant gains in trunk control, balance, and gross motor function [16].

In addition, a recent RCT comparing neurodevelopmental and video game–based trunk training in children with unilateral CP showed that all interventions improved trunk control and balance, with no protocol proving clearly superior. These findings suggest that trunk-focused strategies can be flexibly adapted to individual needs and clinical contexts [21]. A 2021 systematic review of 15 RCTs also concluded that trunk-targeted interventions are effective for improving trunk control, balance, and gross motor function. However, it did not assess spasticity and excluded several recent trials, limiting its comprehensiveness [22].

Despite the clinical relevance of these findings, most existing studies were conducted in single centers and have not been integrated into a comprehensive meta-analysis. Moreover, previous reviews often examined outcomes in isolation, thereby overlooking the interrelated roles of trunk control and spasticity in overall functional performance.

Therefore, this study aims to systematically review and meta-analyze randomized controlled trials that examine the effects of trunk-centered interventions—such as core stability training, neurodevelopmental exercises, and task-oriented therapies—on four key outcomes in children with cerebral palsy: trunk control, gross motor function, balance, and spasticity. This comprehensive synthesis seeks to address prior gaps in the literature by integrating functionally interrelated outcomes and offering evidence-based guidance for clinical neurorehabilitation.

## 2. Materials and Methods

A systematic review and meta-analysis were conducted in accordance with the PRISMA (Preferred Reporting Items for Systematic Reviews and Meta-Analyses) statement and guidelines [23,24]. The protocol for this review was registered in the PROSPERO International Prospective Register of Systematic Reviews (CRD42023418179).

### 2.1. Search Strategy

A comprehensive literature search was conducted by two independent reviewers (MS-L and BC-Y) across five electronic databases—PubMed, Embase, MEDLINE, Web of Science, and CINAHL—for randomized controlled trials (RCTs) published in English between 1 January 2013, and 11 April 2023. The search strategy was developed using the PICO framework, targeting children and adolescents with cerebral palsy (Population), trunk-focused exercise programs (Intervention), non-trunk-specific or conventional therapy (Comparison), and outcomes related to trunk control, gross motor function, balance, and spasticity (Outcomes).

Search terms included combinations of Medical Subject Headings (MeSH) and free-text keywords: “Cerebral Palsy,” “Trunk,” “Core Stability,” and “Randomized Controlled Trial.” Boolean operators (AND/OR) were used to refine the queries. For example, a representative search string used in PubMed was: (“Cerebral Palsy” [MeSH] AND (“Trunk” OR “Core Stability”) AND “Randomized Controlled Trial” [Publication Type]).

After duplicates were removed, titles and abstracts were screened independently by the two reviewers. Full-text articles of potentially eligible studies were assessed according to predefined inclusion and exclusion criteria. Disagreements during any stage of the screening process were resolved through discussion with a third reviewer (HW-L). Only studies published in English were included, due to feasibility constraints and the lack of translation resources.

### 2.2. Inclusion and Exclusion Criteria

Studies were included if they met the following criteria: (1) Included children and adolescents aged 0 to 18 years diagnosed with CP; (2) Evaluated the effectiveness of trunk-focused interventions using at least one relevant outcome measure (e.g., trunk control, gross motor function, balance, or spasticity); (3) Were randomized controlled trials; (4) Reported sufficient statistical data (e.g., sample size, mean, and standard deviation); (5) Were published in English within the past 10 years (from 1 January 2013 to 11 April 2023). Studies were excluded if they met any of the following criteria: (1) Focused on passive interventions (e.g., orthoses, seating aids); (2) Involved surgical procedures; (3) Focused on water-based or alternative exercise therapies (e.g., aquatic therapy, hippotherapy, garment therapy), due to their multimodal and generalized effects, limited trunk-specific targeting, and heterogeneity in intervention protocols; (4) Did not report sufficient statistical data for analysis.

### 2.3. Data Extraction

A total of fifteen studies met the inclusion and exclusion criteria and were included in the final analysis. The search and selection process was documented using the PRISMA flow diagram. Data were systematically extracted using a standardized Excel worksheet (Microsoft Inc., Redmond, WA, USA).

The extracted information included the number of participants in the control and experimental groups, study design and duration, outcome measures, and summary results. Outcome data were extracted as the mean, standard deviation, and sample size for both the control and experimental groups before and after the intervention.

### 2.4. Risk of Bias and Methodological Quality Assessment

The methodological quality of the included randomized controlled trials (RCTs) was assessed using the PEDro scale (https://pedro.org.au/, accessed on 11 April 2023). Two reviewers (MS-L. and BC-Y.) independently evaluated all included studies, including two not listed in the PEDro database, to ensure consistency and completeness. The PEDro scale consists of 11 items, of which the first (eligibility criteria) is not scored. The remaining 10 items are rated as “Yes” (1 point) or “No” (0 points), producing a maximum possible score of 10. Studies were classified as poor (<4 points), fair (4–5 points), good (6–8 points), or excellent (9–10 points) based on their total scores.

In addition to methodological quality, the risk of bias was assessed using the Cochrane Risk of Bias (RoB) tool. Two reviewers (MS-L. and BC-Y.) independently assessed each of the seven standard domains: random sequence generation, allocation concealment, blinding of participants and personnel, blinding of outcome assessment, incomplete outcome data, selective reporting, and other sources of bias. Disagreements were resolved through discussion with a third reviewer (HW-L.). Each domain was judged as presenting a low, high, or unclear risk of bias based on the information provided in the original studies.

To evaluate the overall certainty of the evidence for each outcome, the Grading of Recommendations Assessment, Development and Evaluation (GRADE) approach was applied. As only RCTs were included, the initial quality of evidence for each outcome began as “high” and was downgraded by one level based on the following predefined criteria:

First, the risk of bias domain was downgraded when more than 50% of the contributing studies had a PEDro score below 6, indicating low methodological quality. Second, inconsistency was identified when substantial heterogeneity was present, as indicated by an I^2^ value exceeding 50%. Third, indirectness was considered when the evidence did not directly address the population or intervention of interest—such as when participants were not children with cerebral palsy or the intervention was not specifically trunk-focused. Fourth, imprecision was determined by wide confidence intervals (e.g., ≥0.8 in standardized mean difference) or insufficient total sample size. Finally, publication bias was considered likely when asymmetry was observed in the funnel plot, suggesting the presence of reporting bias.

The final GRADE ratings were categorized as high, moderate, low, or very low, and used to interpret the strength of evidence for each outcome in this meta-analysis.

### 2.5. Statistical Analysis

Standardized mean differences (SMDs) and 95% confidence intervals (CIs) were calculated using RevMan version 5.3 (The Cochrane Collaboration), employing the inverse variance method within either a fixed-effects or random-effects model, depending on the degree of heterogeneity.

Meta-analyses were conducted when at least two studies reported the same outcome variable with available quantitative data before and after the intervention. When outcome measures were assessed using different instruments or scales, the SMD was calculated. If the same instrument and units were used across studies, the mean difference (MD) was applied instead.

A fixed-effects model was used when heterogeneity was negligible; otherwise, a random-effects model was applied. Heterogeneity was assessed using the I^2^ statistic and interpreted as follows: 0–25% (low), 26–75% (moderate), and >75% (high heterogeneity).

Publication bias was evaluated through visual inspection of funnel plots. Effect sizes were interpreted according to Cohen’s criteria: small (SMD = 0.20–0.49), medium (0.50–0.79), and large (≥0.80) [25]. Forest plots were used to display the direction and magnitude of effect sizes with corresponding 95% CIs. A two-sided *p*-value of <0.05 was considered statistically significant.

## 3. Results

### 3.1. Search Results

A total of 211 studies were initially identified through database searching. After removing 21 duplicates, 190 records remained for title and abstract screening. Subsequently, full-text articles were reviewed to assess eligibility based on the predefined inclusion and exclusion criteria. Of these, 175 studies were excluded for the following reasons: 76 employed interventions inconsistent with the scope of this review; 4 lacked sufficient statistical data; 1 was not published in English; 13 did not meet the study design criteria; 78 involved ineligible participants; and 3 reported outcome measures misaligned with the study objectives.

**Figure 1:**
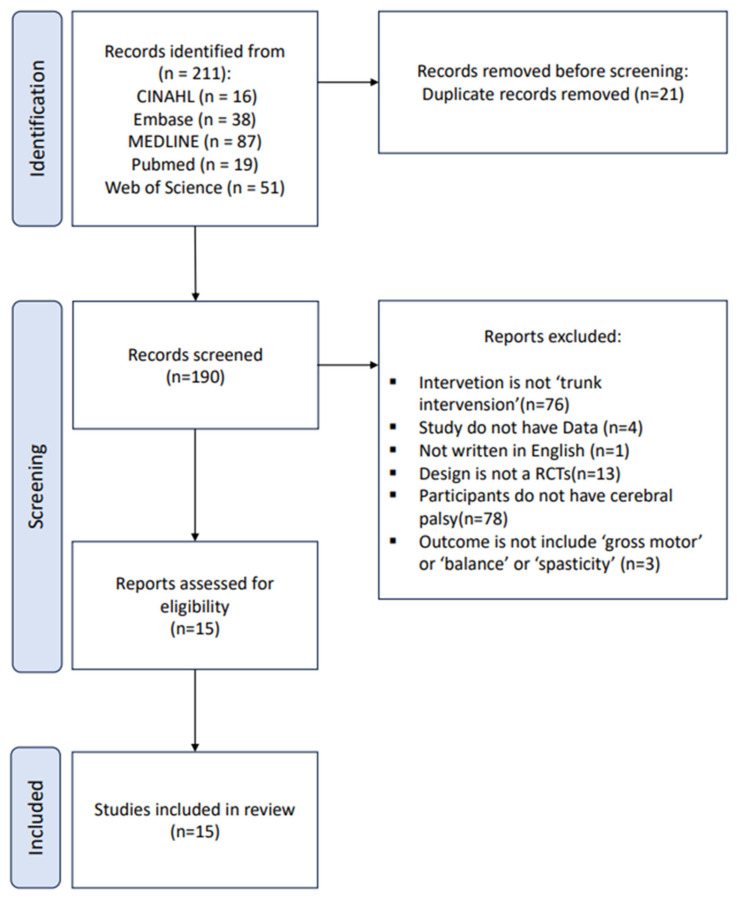
Flowchart of the search and study selection process.

Ultimately, 15 randomized controlled trials met the eligibility criteria and were included in the systematic review. Of these, 9 were included in the meta-analysis across three domains: trunk control (*n* = 5), gross motor function (*n* = 5), and balance (*n* = 4), with some studies contributing to multiple outcome categories. The study selection process is illustrated in the PRISMA flow diagram (Figure 1).

### 3.2. Characteristics of Included Studies

Fifteen randomized controlled trials (RCTs) [5,16,17,19,26,27,28,29,30,31,32,33,34,35,36], published between 2016 and 2023, met the inclusion criteria. Among these, three studies were conducted in Turkey [5,19,27], one in the United Arab Emirates (Dubai) [26], two in India [16,28], four in Egypt [17,29,30,35], three in Pakistan [31,33,34], and two in South Korea [32,36].

A total of 452 participants completed the interventions, with individual study sample sizes ranging from 13 [36] to 44 [16]. Participants were children and adolescents aged between 4 and 18 years. Diagnoses included various types of cerebral palsy, such as spastic diplegia, spastic quadriplegia, spastic hemiplegia, ataxia, and hypotonia. Functional severity was classified using the Gross Motor Function Classification System (GMFCS) levels I to IV.

The key characteristics of the included studies, including country of origin, study design, participant details, intervention types, and outcome measures, are summarized in Table 1.

### 3.3. Evaluation of Study Quality

The methodological quality of the 15 included studies was assessed using the PEDro scale. One study (6.6%) scored 3 points, one (6.6%) scored 4 points, five (33.3%) scored 5 points, four (26.6%) scored 6 points, and four (26.6%) scored 7 points. The mean PEDro score across all studies was 5.6 (Table 1).

**Figure 2:**
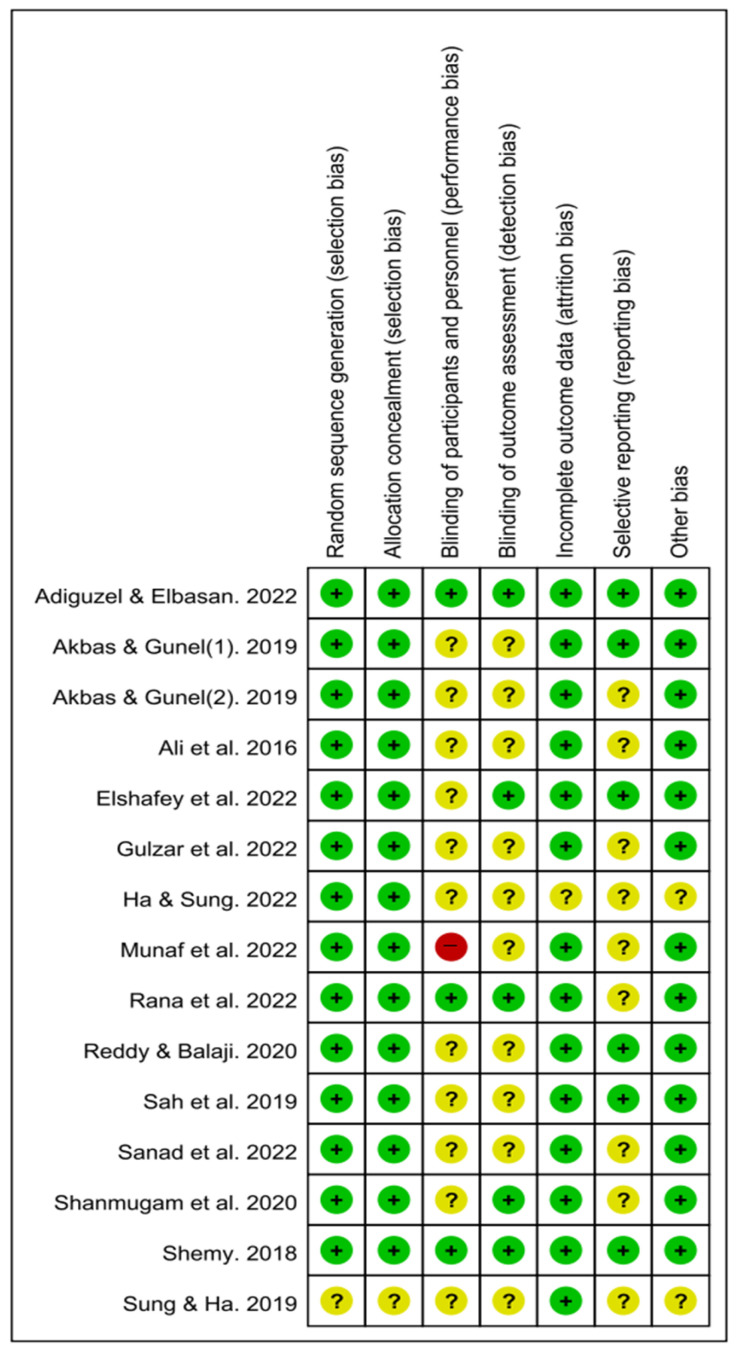
Risk of bias summary for included studies. Review authors’ judgments about each risk of bias item for each included study. “+” indicates low risk, “−” indicates high risk, and “?” indicates unclear risk of bias across the seven Cochrane domains [5,16,17,19,27,28,29,30,31,32,33,34,35,36].

Risk of bias was evaluated across seven domains following the Cochrane Risk of Bias (RoB) guidelines, and the results are summarized in Figure 2. For random sequence generation, 14 studies (93.3%) were rated as having a low risk of bias, while 1 study (6.6%) was rated as having an unclear risk. For allocation concealment, 14 studies (93.3%) were judged to be at low risk and 1 (6.6%) at unclear risk.

Regarding blinding of participants and personnel, 3 studies (20.0%) were assessed as low risk, 1 (6.6%) as high risk, and 11 (73.3%) as unclear. For blinding of outcome assessment, 5 studies (33.3%) were judged to be at low risk and 10 (66.6%) at unclear risk.

In the domain of incomplete outcome data, 14 studies (93.3%) were rated as low risk and 1 (6.6%) as unclear. For selective reporting, 6 studies (40.0%) were assessed as low risk, while 9 (60.0%) were categorized as unclear. Finally, in the domain of other sources of bias, 13 studies (86.6%) were rated as low risk and 2 (13.3%) as unclear.

### 3.4. Effects of Trunk Intervention

#### 3.4.1. Trunk Control

Eight of the fifteen included studies assessed trunk control using validated outcome measures. These included the Trunk Impairment Scale (TIS; *n* = 2), Trunk Control Measurement Scale (TCMS; *n* = 3), Segmental Assessment of Trunk Control (SATCo; *n* = 1), trunk angle and sway in the sitting position (*n* = 1), and trunk endurance (*n* = 1). All studies reported statistically significant improvements in trunk control in the intervention groups compared to the control groups.

The pooled effect size from the three studies that used the TCMS was not statistically significant (SMD = 2.24; 95% CI: −0.22 to 4.69; I^2^ = 0%). In contrast, the two studies utilizing the TIS showed a statistically significant and large effect size (SMD = 3.76; 95% CI: 3.17 to 4.35; I^2^ = 0%).

**Figure 3:**
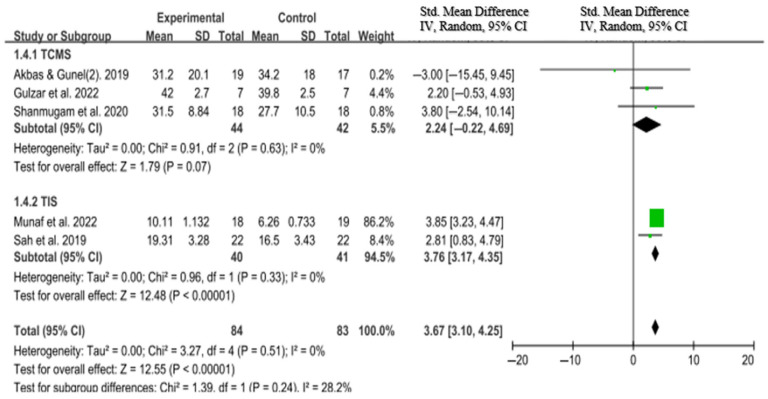
Forest plot of the effect of trunk-focused interventions on trunk control in children with CP [16,19,26,31,33].

When data from all five studies using standardized outcome measures (TCMS and TIS) were combined, the overall pooled effect size remained statistically significant and large (SMD = 3.67; 95% CI: 3.10 to 4.25; I^2^ = 0%), indicating substantial improvement in trunk control following trunk-focused interventions (Figure 3).

#### 3.4.2. Gross Motor Function

Seven studies assessed gross motor function: six used the Gross Motor Function Measure (GMFM), and one employed the Bruininks–Oseretsky Test of Motor Proficiency, Second Edition (BOT-2). All studies reported statistically significant improvements in the intervention groups compared to the control groups. Six GMFM-based studies were included in the meta-analysis.

A subgroup analysis was conducted based on the frequency of intervention. One study that did not specify intervention frequency was excluded, resulting in five studies analyzed. For interventions conducted more than four days per week, the pooled effect size was small but statistically significant (SMD = 0.49; 95% CI: 0.02 to 0.95; I^2^ = 0%). In contrast, for interventions provided fewer than three days per week, the pooled effect size was not statistically significant (SMD = 0.47; 95% CI: −0.33 to 1.28; I^2^ = 70%), likely due to the wide confidence interval and substantial heterogeneity.

**Figure 4:**
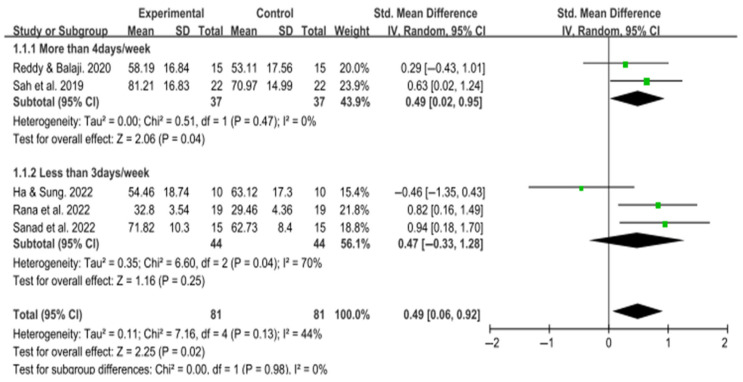
Forest plot of the effect of trunk-focused interventions on gross motor function in children with CP [16,28,32,34,35].

The overall pooled effect size across all included studies was small but statistically significant (SMD = 0.49; 95% CI: 0.06 to 0.92; I^2^ = 44%), indicating that trunk-focused interventions resulted in modest improvements in gross motor function among children with cerebral palsy (Figure 4).

#### 3.4.3. Balance

Eight studies assessed balance using a range of outcome measures, including the Pediatric Balance Scale (PBS; 5 studies), Balance Error Scoring System (BESS; 1 study), HUMAC Balance System (1 study), Biodex Balance System (1 study), Five Times Sit-to-Stand Test (FTSTS; 1 study), Timed Up and Go (TUG; 1 study), and the Pediatric Berg Balance Measure (PBBM; 1 study). All studies reported significant improvements in balance in the intervention groups compared to the control groups.

Of these, five studies using the PBS were included in the meta-analysis. A subgroup analysis was conducted based on intervention frequency. One study lacking frequency information was excluded, leaving four studies for analysis.

For interventions administered more than four days per week, the pooled effect size was moderate and statistically significant (SMD = 0.54; 95% CI: 0.08 to 1.01; I^2^ = 0%). In contrast, for interventions conducted fewer than three days per week, the pooled effect size was large but not statistically significant (SMD = 1.29; 95% CI: −0.86 to 3.44; I^2^ = 90%), likely due to substantial heterogeneity and wide confidence intervals.

**Figure 5:**
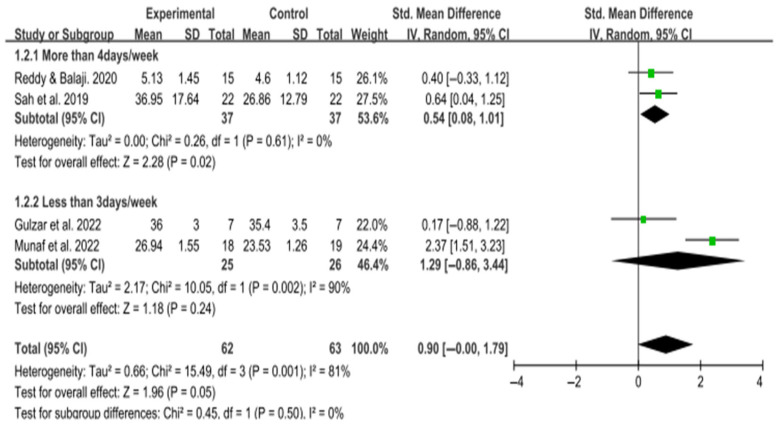
Forest plot of the effect of trunk-focused interventions on gross motor function in children with CP [16,28,31,33].

The overall pooled effect size across all PBS-based studies was large but did not reach statistical significance (SMD = 0.90; 95% CI: −0.00 to 1.79; I^2^ = 81%), suggesting that trunk-focused interventions may improve balance. However, further high-quality studies with standardized intervention protocols are needed to confirm these effects (Figure 5).

#### 3.4.4. Spasticity

Only one study assessed spasticity using the Modified Tardieu Scale (MTS). The results indicated no statistically significant differences in spasticity between the intervention and control groups following the treatment.

## 4. Discussion

A total of 15 studies, including 454 children with cerebral palsy (CP), systematically evaluated the effects of trunk-targeted interventions on motor function, balance, and spasticity. The results showed that children in the experimental groups achieved significantly better outcomes than those in the control groups across these domains. These findings suggest that trunk-focused interventions may significantly enhance motor function and balance, and potentially reduce spasticity in children with CP. Notably, treatment duration appeared to be a key factor influencing the observed improvements.

All eight studies that specifically assessed trunk control reported significant improvements following trunk-targeted interventions. Of these, five studies were included in the meta-analysis, which revealed a very large effect size (3.67), reflecting considerable improvements in trunk endurance and muscle activation. For instance, El-Shemy evaluated the effects of core stabilization training on trunk endurance in children with cerebral palsy using standardized trunk endurance tests. The control group showed improvements only in trunk extensor endurance, while the experimental group exhibited significant gains in flexor, extensor, and bilateral lateral trunk muscle endurance [17]. Similarly, Munaf et al. [33] combined trunk exercises with conventional physical therapy and reported notable gains in trunk control, balance, and mobility in the experimental group.

Trunk-focused interventions based on neurofacilitation approaches—including neurodevelopmental treatment (NDT), proprioceptive neuromuscular facilitation (PNF), and Vojta therapy—also demonstrated effectiveness in enhancing postural control and trunk stability [5,19,26,32,36]. In particular, Vojta therapy was associated with increased thickness of the rectus abdominis and external oblique muscles, suggesting the critical role of these muscles in achieving proximal trunk stability [36]. A study comparing modified Pilates and NDT showed greater improvement with Pilates, likely due to more targeted core strengthening [27]. Likewise, functional training programs that included activities such as gym ball exercises, treadmill walking, and cycling also demonstrated comparable or superior functional gains relative to lower-limb-focused training [31].

All seven studies assessing gross motor function reported significant improvements following intervention. Among these, five studies were included in the meta-analysis, which revealed a small but clinically meaningful effect size (SMD = 0.49). Although the effect size appears modest numerically, it falls within the minimum clinically important difference (MCID) range of 0.1–3.0 points on the GMFM-88, as defined by Storm et al. [37]. affirming the clinical relevance of trunk-focused interventions. Therefore, the observed effect size (SMD = 0.49) may be interpreted as corresponding to a GMFM-88 improvement within this MCID range, reinforcing the clinical relevance and therapeutic value of trunk-focused interventions.

Studies based on neural facilitation models—such as NDT and proprioceptive strategies—also reported improved gross motor function and enhanced performance in daily living activities [30,34,35]. For example, Reddy and Balaji utilized dynamic surface exercises that stimulated multiple sensory systems, including visual, proprioceptive, and mechanoreceptive inputs. These exercises enabled better vestibular feedback and postural awareness, ultimately improving trunk control and gross motor function [28]. These exercises stimulated visual, proprioceptive, and mechanoreceptive inputs. This multisensory stimulation enabled participants to perceive head orientation through vestibular feedback and detect support surface direction via proprioception, ultimately improving trunk control and gross motor outcomes. Three studies in the review specifically evaluated core stability and trunk stabilization exercises, all of which reported statistically significant enhancements in gross motor function [30,34,35]. Core strengthening helps stabilize the lumbar and pelvic regions, thereby improving axial postural control during gait [35]. Marshall et al. [38] further emphasized that core stability training enhances lumbar and pelvic alignment and reinforces associated musculature, leading to greater trunk support during limb movements.

In addition, improving motion in restricted spinal segments can influence overall motor performance and mobility. Curtis et al. [39] explored the relationship between trunk segmental control and gross motor function and concluded that trunk control is a key determinant of motor ability. Notably, their study suggested that improving postural control within a single SATCo segment could lead to a GMFM score increase of 0.5 to 11 points. They also identified minimum trunk control thresholds required for progression to higher GMFM dimensions and highlighted trunk control as a distinguishing factor among GMFCS levels.

All eight studies evaluating balance outcomes reported significant improvements following trunk interventions. Among them, four were included in the meta-analysis, which yielded a large effect size (SMD = 0.90). However, statistical significance was not achieved, likely due to small sample sizes or methodological heterogeneity.

Studies implementing core stabilization exercises demonstrated statistically significant improvements in balance, particularly when combined with conventional physiotherapy [18,29]. The core region—encompassing the body’s center of gravity—is crucial for maintaining postural control. Strengthening core muscles minimizes displacement and fluctuations in the center of gravity, enhances neuromuscular efficiency, and provides structural stability for coordinated movement [29]. These exercises also improve anticipatory control mechanisms, stimulate proprioceptive feedback, activate trunk musculature, and enhance somatosensory integration under static and dynamic conditions [30].

Neural facilitation-based interventions such as NDT and PNF have also shown benefits in balance improvement [18,19]. These findings align with motor development principles, where proximal stability supports distal mobility. Enhanced proximal trunk control thereby leads to improved distal limb coordination and postural balance [18]. Dynamic surface exercises designed to improve trunk control also yielded significant improvements in balance [28]. Training on unstable surfaces has demonstrated superior efficacy in increasing trunk muscle cross-sectional area and improving postural control when compared to stable surface exercises [40]. Furthermore, trunk stabilization interventions that actively engage the child have demonstrated effectiveness in improving both static and dynamic balance in children with cerebral palsy [41]. These results collectively support the therapeutic potential of dynamic surface exercise in enhancing balance in this population. Across the included studies, the frequency of balance-focused interventions ranged from three to six sessions per week. Subgroup analysis revealed that interventions delivered more than four times weekly yielded statistically significant improvements with a moderate effect size. In contrast, studies with lower intervention frequency (three sessions or fewer per week) showed high heterogeneity, likely due to variability in total intervention duration (ranging from 8 weeks to 3 months). However, due to inconsistent reporting and methodological heterogeneity, the optimal dosage (i.e., frequency and duration) of trunk-centered interventions could not be clearly identified. Future studies should aim to standardize and report these parameters to facilitate evidence-based recommendations.

Only one study directly investigated the effects of trunk interventions on spasticity and found no statistically significant differences. Akbas and Gunel implemented a trunk training program combining neurodevelopmental treatment and muscle strengthening [5]. They noted that while insufficient trunk control may contribute to abnormal movement patterns, it does not appear to directly affect spasticity. They concluded that trunk exercises grounded in neural facilitation and strengthening principles can be safely applied without increasing or exacerbating spasticity [5].

Similarly, Merino-Andres et al. [42] reported that muscle strengthening exercises did not exacerbate spasticity and were associated with improvements in both gross motor function and balance. Another study similarly observed increased muscle strength following strength training in children with cerebral palsy, without a concomitant rise in spasticity levels [43].

Among the included studies, eight incorporated strength-based components, including core stabilization exercises. None of these reported adverse effects related to spasticity; instead, all demonstrated positive outcomes in gross motor function, balance, or other functional domains. However, as only one study specifically examined the direct relationship between trunk interventions and spasticity, the current evidence base is insufficient to draw definitive conclusions regarding their effect on spasticity.

This review has several limitations. First, it included only studies published in English, which may have introduced language bias and limited the generalizability of the findings. Second, the analysis was restricted to randomized controlled trials (RCTs), thereby excluding potentially valuable insights from high-quality non-randomized studies that also examined trunk interventions. Third, despite employing a comprehensive search strategy, the number of studies included in the meta-analysis was relatively small. This limited sample size may have contributed to an overestimation or underestimation of the true effect sizes and reduced the power to detect subgroup differences.

To overcome these limitations, future research should include studies published in other languages, consider methodologically robust non-RCT designs, and broaden the scope of eligible studies. Such efforts would contribute to a more comprehensive and accurate understanding of the effects of trunk interventions on trunk control, gross motor function, balance, and spasticity in children with cerebral palsy.

## 5. Conclusions

This systematic review and meta-analysis provide comprehensive evidence supporting the efficacy of trunk-focused interventions in improving trunk control, gross motor function, and balance in children with cerebral palsy. The findings highlight the therapeutic importance of enhancing trunk stability as a key component of pediatric neurorehabilitation.

The meta-analytic revealed particularly large effect sizes for trunk control and clinically meaningful improvements in gross motor function and balance. Intervention frequency emerged as a key moderator, with higher-frequency interventions (more than four sessions per week) generally yielding more favorable outcomes.

However, due to the limited number of studies examining spasticity, conclusions regarding its modulation remain tentative. Although no adverse effects were reported, further research is needed to clarify the direct impact of trunk interventions on spasticity.

These findings support the clinical adoption of trunk-centered rehabilitation strategies, including core stability training and task-specific exercises. Emerging technologies—such as virtual reality, motion-sensing games, and robotic-assisted therapy—also show potential for enhancing engagement and treatment precision. Their integration into clinical programs warrants further investigation. Future high-quality studies should refine intervention protocols, establish optimal dosing, and contribute to standardized, evidence-based guidelines for pediatric practice.

## Figures and Tables

**Table 1:** Characteristics of included studies.

Author/Year/PEDro Score	Sample Size (EG/CG)	Participants (CP Type/GMFCS)	Intervention	Duration and Frequency	Outcome Measures
Adıguzel et al./2022 [27]/6/10	EG: 9/CG: 9	CP/GMFCS I–III	EG; Modified Pilates Exercises CG: Neurodevelopmental treatment	8 weeks, 60 min/day, 2 days/week	TCMS, SPCM, PRT, PBBM, 6 MWT, OGS
Akbas et al./2019a [5]/5/10	EG: 19/CG: 17	Bilateral spastic CP/ GMFCS I–V	EG; trunk training CG; routine physiotherapy	8 weeks, 45–75 min/day, 2 days/week	Modified Tardieu Scale, Surface Electromyography
Akbas et al./2019b [19]/4/10	EG: 19/CG: 17	Spastic CP/ Not specified	EG; trunk training CG; routine physiotherapy	8-week, 45 min/day, 2 days/week	GMFM, TCMS, QUEST, PBS, IPFAM, Gillette FAS
Ali et al./2016 [29]/5/10	EG: 15/CG: 15	Spastic diplegia/ Not specified	EG; selective therapeutic exercises + core stabilizing program CG; selective therapeutic exercises	8 weeks, 60 min/day, 3 days/week	Biodex stability system
Elshafey et al./2022 [30]/6/10	EG: 20/CG: 20	Cerebellar ataxic CP/ Not specified	EG; core stability program+ selected physical therapy program CG; standard physical therapy program	8 weeks, 60 min/day, 3 days/week	SARA, BESS, BOT-2
Gulzar et al./2022 [31]/5/10	EG: 7/CG: 7	CP/GMFCS II–III	EG; functional training CG; conventional therapy	8 weeks, 45–60 min/day, 3 days/week	PBS, TCMS, FTSST, TUG
Ha et al./2022 [32]/5/10	EG: 10/CG: 10	Hypotonia/ Not specified	EG; Vojta therapy CG; general physical therapy	6 weeks, 30 min/day, 3 days/week	Abdominal muscle thickness, SATCo, trunk angle and sway (sitting), GMFM
Munaf et al./2022 [33]/7/10	EG: 18/CG: 19	Spastic hemiparetic CP/ GMFCS I–II	EG; conventional physical therapy + trunk exercise CG; conventional physical therapy	12 weeks, 60 min/day, 3 days/week	TIS, PBS, DGI
Rana et al./2022 [34]/6/10	EG: 19/CG: 19	Spastic diplegic CP/ GMFCS II–III	EG; trunk stabilization CG; conventional physiotherap	6 weeks, 45 min/day, 3 days/week	GMFM
Reddy et al./2020 [28]/7/10	EG: 15/CG: 15	Spastic diplegic CP/ GMFCS III–IV	EG; dynamic surface exercise training CG; standard physiotherapy training	6 weeks, 60 min/day, 4 days/week	GMFM, PBS
Sah et al./2019 [16]/7/10	EG: 22/CG: 22	Spastic diplegic CP/ GMFCS II–III	EG; task-oriented activities based on NDT CG; conventional physiotherap	6 weeks, 60 min/day, 6 days/week	GMFM, PAS, PBS, TIS
Sanad et al./2022 [35]/3/10	EG: 15/CG: 15	Spastic diplegia/ GMFCS II–III	EG; core stability exercises Program + NDT/ CG; NDT	8 weeks, 45–60 min/day, 3 days/week	EEI, GMFM
Shanmugam et al./2020 [26]/5/10	EG: 18/CG: 18	Spastic Diplegia/ GMFCS I–III	EG; pelvic PNF + conventional therapy CG; conventional physiotherapy	4 weeks, 30 min/day, 5 days/week	TCMS, PALM
El-Shemy/2018 [17]/7/10	EG: 15/CG: 15	Hemiplegic CP/ GMFCS II	EG; PT program + core stability CG; PT program	8 weeks, 45 min/day, 3 days/week	Trunk endurance tests, gait
Ha et al./ 2020 [36]/6/10	EG: 6/CG: 7	Spastic CP/GMFCS I–III	EG; Vojta approach CG; general exercise	6 weeks, 30 min/day, 3 days/week	abdominal muscles thicknesses, gait

EG: Experimental Group, CG: Control Group, GMFCS: gross motor function classification system, TCMS: Trunk control measurement scale, SPCM: seated postural control measurement, PRT: Pediatric Reach Test, PBBM: Pediatric Beg Balance Measurement, 6MWT: 6 Minute walking Test, OGS: Observational Gait Scale, GMFM: Gross Motor Function Measure, PAS: Postural Assessment Scale, PBS: Pediatric Balance Scale, TIS: Trunk Impairment Scale, QUEST: Quality of Upper Extremity Skills Test, IPFAM: Impact on Family Scale, Gillette FAS: Gillette Functional Assessment Scale, SARA: Scale for the assessment and rating of ataxia, BESS: Balance Error Scoring Systems Scale, BOT-2: Bruininks–Oseretsky Test of Motor Proficiency, FTSST: Five Times Sit to Stand Test, TUG: Timed-Up-and-Go Test, SATCo: Segmental Assessment of Trunk Control, DGI: Dynamic Gait Index Scale, PAS: postural assessment scale, TIS: trunk impairment scale, PALM: Palpation Meter Device, EEI: Energy Expenditure Index.

## Data Availability

All data are available within the manuscript and can be obtained from the corresponding author upon reasonable request.
